# Perspective of German medical faculties on digitization in the healthcare sector and its influence on the curriculum

**DOI:** 10.3205/zma001520

**Published:** 2021-11-15

**Authors:** Maximilian Neumann, Leonard Fehring, Kristian Kinscher, Hubert Truebel, Florian Dahlhausen, Jan P. Ehlers, Thomas Mondritzki, Philip Boehme

**Affiliations:** 1 Witten/Herdecke University, Faculty of Health, School of Medicine, Witten, Germany; 2 Bayer Pharma AG, Cardiovascular Research, Wuppertal, Germany

**Keywords:** medical education, German medical faculties, digitalization, upskilling

## Abstract

**Background: **The German healthcare sector is in the process of being disrupted by digitization. Universities are asked to reflect on the consequences and develop strategies to prepare their medical students for a digitalized health care sector. The current state of research does not systematically record the associated activities of individual medical faculties in Germany.

**Objective: **This study was designed to survey the status-quo of how German medical faculties view the digitization progress and to what extend digital capability building is already integrated into the curricula.

**Methods: **A questionnaire with three focus areas was developed: Firstly, the general view of the medical faculties on digitization; secondly, concrete measures to prepare students for digital change and thirdly, the overarching organizational and regulatory conditions. The data was collected through short, questionnaire-based telephone interviews among those responsible for the curriculum at their faculty. The datasets collected were anonymized and statistically evaluated.

**Results: **30 interviews were conducted. The majority of faculty representatives agreed that digitization will change the role of physicians (87% agreement). Changes caused by individual digitization trends were however viewed to be less likely, e.g., whether medical expertise will become less important due to digital assistance systems (20% agreement), whether physician positions will be replaced by robots and algorithms (7% agreement), or whether hierarchies in hospitals will flatten (13% agreement). Digitization was seen to be of major importance for medical studies (93% agreement). Associated content should be given a higher priority in the curriculum (87% agreement). Two-thirds of faculty representatives believed that overarching institutions such as politics and medical associations ought to have more concrete plans for implementing the digital transformation and that innovations should be implemented in practice faster.

**Conclusion: **While most faculty representatives attach great importance to the digitization of the health care system for university education, various questions about structural teaching measures to prepare students for the digital change show that there is no uniform education of medical students for a digitized health care system. We were also able to show that most faculty representatives are dissatisfied with the regulatory and organizational conditions of digitization in the medical sector.

## Zusammenfassung

**Hintergrund: **Das Gesundheitswesen in Deutschland befindet sich im Prozess der Digitalisierung. Für Universitäten stellt sich daher die Frage, wie sie ihre Medizinstudierenden auf einen digitalisierten Gesundheitssektor vorbereiten sollten. Der aktuelle Forschungsstand weist hierzu bisher keine systematische Erfassung der Aktivitäten deutscher Medizinfakultäten auf.

**Zielsetzung: **Ziel dieser Studie war es systematisch zu erfassen, wie deutsche Medizinfakultäten den Einfluss und Fortschritt der Digitalisierung beurteilen und inwieweit die Vorbereitung der Studierenden auf ein „digitaleres“ Gesundheitssystem bereits heute in den Curricula berücksichtigt wird.

**Methodik: **Ein strukturierter Fragebogen wurde entwickelt. Drei Themen waren dabei im Fokus: Die Sicht der Fakultäten auf die Digitalisierung des Gesundheitswesens insgesamt, deren Maßnahmen zur Vorbereitung der Studierenden auf den digitalen Wandel im Lehrplan und die übergreifenden, organisatorischen Rahmenbedingungen. Die Daten wurden mit Hilfe des Fragebogens in kurzen Telefoninterviews mit den für den Lehrplan Verantwortlichen verschiedener medizinischer Fakultäten erhoben. Die erhobenen Datensätze wurden anonymisiert und systematisch statistisch ausgewertet.

**Ergebnisse: **Es wurden 30 Interviews geführt. Die Fakultätsvertreter stimmten mehrheitlich zu, dass Digitalisierung die Rolle von Ärztinnen und Ärzten verändern wird (87% Zustimmung). Individuelle Digitalisierungstrends wurden als unwahrscheinlicher eingeschätzt, z. B. ob Fachwissen durch digitale Assistenzsysteme unwichtiger wird (20% Zustimmung), ärztliche Stellen durch Roboter und Algorithmen ersetzt werden (7% Zustimmung) oder Hierarchien in Krankenhäusern flacher werden (13% Zustimmung). Digitalisierungsinhalten wurde eine wesentliche Bedeutung für das Medizinstudium beigemessen (93% Zustimmung). Diese sollten einen höheren Stellenwert im Curriculum erhalten (87% Zustimmung). Zwei Drittel der Fakultätsvertreter waren der Meinung, dass übergreifende Institutionen wie Ärztekammern und Politik konkretere Pläne zur Umsetzung der digitalen Transformation benötigen und eine schnellere Implementierung von Innovationen in der Praxis notwendig sei.

**Schlussfolgerung: **Während die Mehrheit der Fakultätsvertreter der Digitalisierung des Gesundheitssystems eine hohe Bedeutung für die universitäre Ausbildung beimisst, zeigen verschiedene Fragen nach strukturellen Lehrmaßnahmen zur Vorbereitung der Studierenden auf den digitalen Wandel, dass es keine einheitliche Ausbildung von Medizinstudierenden für ein digitalisiertes Gesundheitssystem gibt. Außerdem konnten wir zeigen, dass die Mehrheit der Fakultätsvertreter mit den regulatorischen und organisatorischen Rahmenbedingungen für die Digitalisierung im medizinischen Sektor unzufrieden ist.

## 1. Introduction

Digitization is often credited with enormous transformational power. Through the transformation of analogue information into digital form and resulting societal effects, it has led to far-reaching changes in nearly all economic sectors in recent years [1], [2]. In health care, this process – here conceptualized as the use of information and communication technologies to support clinical practice – has developed slower than in almost all other economic sectors. Between 1991 and 2013, digital progress in the social and health care sector was just one percent compared to 25-30% in other sectors [3]. 

The speed of this digitization progress has been particularly slow in Germany. A 2018 study by the Bertelsmann Foundation examined the extent of digitization in the health care system across 17 countries. While Estonia was in first place with a digital health index of over 80 out 100, Germany ranked second last with 30 points [4].

The reasons for this are manifold: Germany’s health care system is highly fragmented. Both at the process level and at the financing level, a large number of interest groups are involved, which makes decision-making challenging [3]. Additionally, data protection is interpreted more strictly in Germany than in other countries [3], [5]. Moreover, Germany’s self-regulatory bodies and the medical profession were found to lack willingness and necessary organizational structures to pursue digitization [6]. This has frequently been attributed to lack of evidence and insufficient interoperability, whereas financial incentives seemed to play a smaller role [7], [8].

In a recent article, Kuhn and colleagues examined these challenges for digitization of the medical profession more closely. They concluded that inadequate training in digital competencies is a key challenge, which begins already at the level of the curriculum. Neither Germany’s National Competency-based Learning Objectives Catalogue (“Nationaler Kompetenzbasierter Lernzielungskatalog”) adopted in 2015 nor the Master Plan for Medical Studies 2020 (“Masterplan Medizinstudium 2020”) contain sufficient plans to prepare students for a digitally transformed health care system [9]. 

This is highly problematic. Firstly, some areas of the health care system have already become increasingly digital, from the introduction of digital patient records to cognitive computing systems for physician decision-making. Secondly, digital change in the health care system has been accelerating over the last years [7], [10]. This has been further intensified by the current COVID-19 pandemic, as for instance highlighted by the increased relevance and rapid adoption of telehealth solutions [11].

This leads to the question of how well future doctors and other health care professionals are prepared for such digital transformation while going through the medical curriculum. Addressing this question, the object of this research was to investigate how medical faculties in Germany perceive digitization and how well they prepare their students for a digitized health care system, and the challenges that may arise.

## 2. Methods

### 2.1. Design 

Based on a literature research and exploratory sample interviews, a preliminary questionnaire was developed. An expert panel with a multi-professional background revised this first questionnaire. The questionnaire focused on three areas:

- General view of medical faculties on digitization of the health care system
- Integration of measures to prepare students for digitization into the medical curriculum
- Assessment of how medical faculties evaluate the overarching organizational conditions.

The preliminary questionnaire was piloted with 5 randomized participants (who were subsequently not included in the results) and then again reviewed and revised by the expert panel. Participants were asked to rate their agreement to 17 statements. We used a numeric analogue Likert-scale of 0 (lowest agreement) to 10 (highest agreement). Studies suggest item-non-response rates to be lower with such scale [12]. Besides the 17 questions using the Likert-Scale and three yes/no questions, we also included an open question that investigated faculties’ plan to prepare the students for the digital change. 

#### 2.2. Recruitment of participants

We contacted the faculties’ offices of all 42 medical faculties in Germany. To ensure representativeness of our data, we always asked for an interview with the dean or most senior academic individual responsible for the medical curriculum development. No further exclusion criteria or screening questionnaires were applied. During the interview, all participants were informed about the study details, including the duration of the interview and data storage policy. The questionnaire was attached to the digital interview request for prior consultation. 

#### 2.3. Data collection and analysis

During the interview, the questionnaire was filled in by the interviewer and confirmed by the interviewee afterwards. All data were subsequently anonymized. The expert panel excluded one question about digital educational methods after evaluation because of a possible misinterpretation that became apparent after several of the interviews had already been performed. Data was analyzed using descriptive statistics in Microsoft Excel (Version 16.38). Response values above 5 (on a 10-point Likert scale) were classified as agreement with the question prompt. 

## 3. Results

A total of 30 participants from 28 public and 2 private universities were interviewed. The faculty representatives we interviewed were 73% male and 27% female. 

### 3.1. General views toward the digitization of the health care system

Most participants (87%) were convinced that digitization will change the role of physicians over the next 10 years (median agreement 9). Only 20% of respondents believed that medical expertise will become less relevant for physicians in the future (median 2). Equally, only a minority of respondents (7%) believed that digital technologies – more specifically artificial intelligence and robotics – will replace doctors (median 2). 

Instead, nearly all participants (97%) believed that the personal contact between doctors and patients will remain vital (median 10). Such a human-centric approach is also highlighted by the fact that over two thirds of participants (71%) believed that doctors find it difficult to transfer core competencies to digital assistance systems (median 7). 

**Figure 1:**
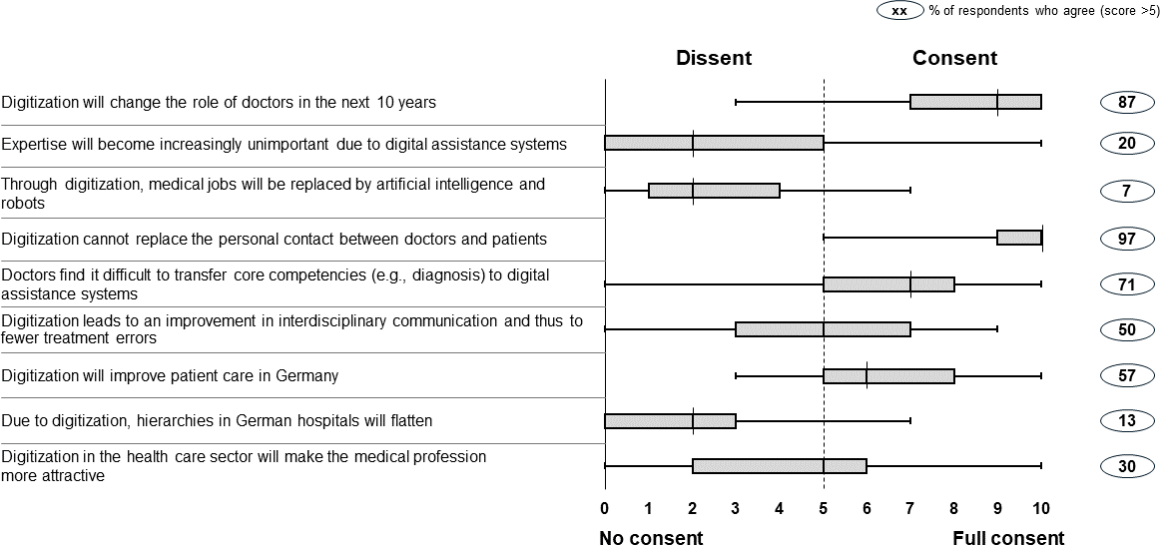
How German medical faculties view the digitization of the health care sector

While participants were undecided whether digitization will result in fewer treatment errors due to better interdisciplinary communication (median 5), 57% agreed that patient care is positively impacted by digitization (median 6). Hierarchies in hospitals were not expected to flatten due to digitization (median 2). Only 30% of participants believed that the medical profession will become more attractive due to digitization (median 5). The results are shown in figure 1 (Fig. 1).

#### 3.2. Integration of digitization into university curricula

Digitization was seen as an essential element of the medical education of students by 93% of participants (median 8). 87% of participants responded that digitization should play a more central role in university curricula (median 8).

**Figure 2:**
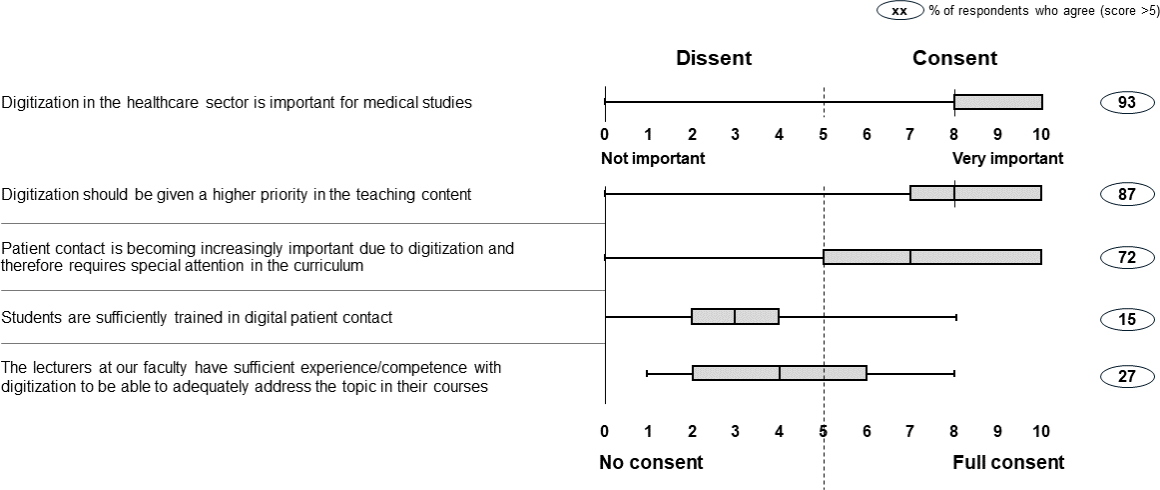
How medical faculties train their students in digitization of the health care sector in Germany

The extent to which faculties have incorporated digitization into their medical curricula varies. 72% faculty representatives believed that patient contact will become more important in light of digitization and should therefore receive special attention in the curriculum (median 7). Yet, only 15% of faculty representatives believed that they train students sufficiently in digital patient contact (median 3). Only 27% of participants believed that professors had the relevant competencies to educate students on digital capabilities (median 4). The results are shown in figure 2 (Fig. 2).

**Figure 3:**
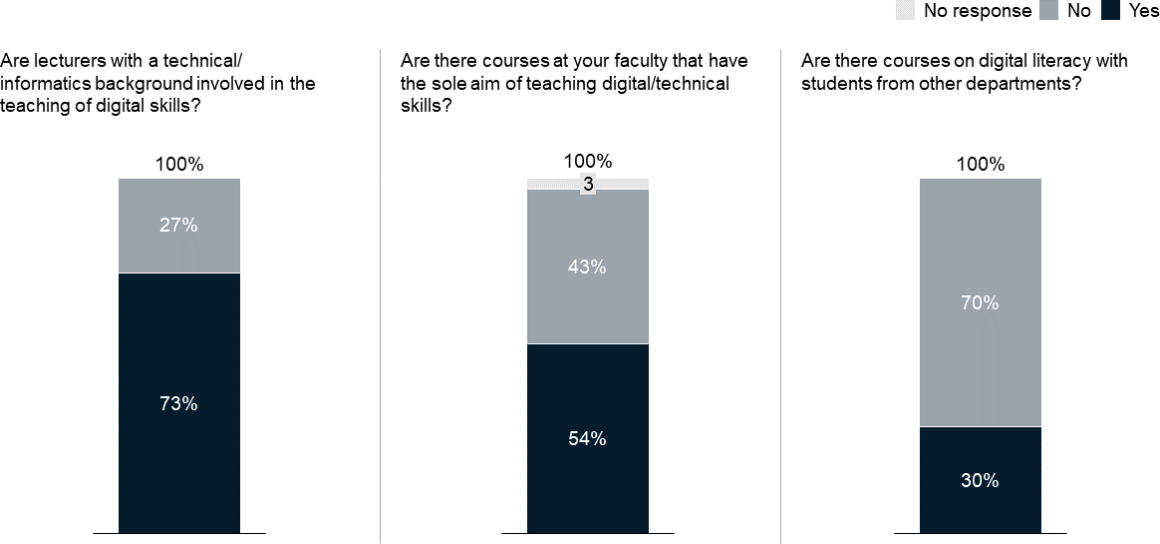
How faculties’ digital education is setup organizationally

A majority of faculty representatives (73%) employs lecturers with technical or computer science backgrounds to teach digital literacy. Every second faculty (54%) offers courses with the sole aim of teaching digital literacy. In 30% of faculties, such digital literacy courses are also attended by students from other faculties. The results are shown in figure 3 (Fig. 3). 

Regarding faculties’ plan to prepare students for the digital change, our open question revealed that only a small minority of faculties (10%) still has to develop a concrete plan to prepare students for the digital change. Other faculties have already implemented or plan to introduce curricular measures such as elective courses for big data, e-health and telemedicine with a multi-disciplinary teaching team, offering students ethical, psychological and economic perspectives on digitization. Faculty representatives indicated that several professorships for medical informatics or data literacy have already been established or will be in the near future. 

#### 3.3. Overarching organizational conditions

**Figure 4:**
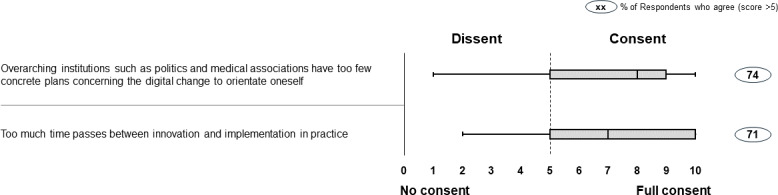
How German medical faculties evaluate the overarching conditions for the digitization of the health care sector

In addition to the questions regarding digitization of health care and medical education, the questionnaire also contained two questions about the regulatory conditions that influence the digital transformation of the health care system. Here, we found a majority of faculty representatives to be dissatisfied with the overarching conditions that we surveyed. With a median of 8, most faculty representatives believed that there are too few overarching concrete plans preparing for the digital change. Moreover, they were convinced that too much time passes between innovation and its implementation in practice (median 7). The results are shown in figure 4 (Fig. 4).

## 4. Discussion

This study aimed to establish an overview of how medical faculties in Germany view the digitization of the health care sector and how they prepare their students for the digital challenges they will face when entering the health care sector as physicians. 

We found that an overwhelming share of faculties believes that digitization will transform the health care sector and the role of physicians in the future. More concrete, individual digitization trends that may cause such transformation were however deemed to be relatively unrealistic, such as a substitution of medical capabilities by artificial intelligence or robotics (see figure 1 (Fig. 1)).

This discrepancy has been reported in the literature numerous times. Often, the overall importance and the benefit of medical informatics is widely acknowledged despite a sometimes unclear understanding of medical informatics and digitization itself. This has been rated as one of the main reasons why such topics played only a minor role in the medical curriculum [13], [14].

Regarding faculties’ preparation of students for the digital change, we found a very heterogeneous picture of the medical curricula landscape. At some faculties, digitization is already an established part of the curriculum, whereas other faculties prepare their students little or not at all for digitization in health care (see figure 2 (Fig. 2) and figure 3 (Fig. 3)). Yet, despite these efforts, broader curricular efforts to prepare future physicians for digitization are needed.

This result is related to findings of various other scholars who have equally called for such change. For instance, Kuhn et al. found the ability to interpret medical data to be insufficiently represented in today’s medical curricula, although one of the most important skills for future physicians [15]. This seems to be an international phenomenon as McGowan et al., who conducted a similar survey like ours in the US, show that only a few schools taught data literacy to evaluate medical information [16]. A similar picture is presented for Australian medical faculties by Edirippulige et al. [14]. The authors interviewed participants from all 19 medical schools in Australia, finding consensus that although the preparation of students for the digital transformation is important, other priorities and systematic problems such as a "crowded" curriculum have so far hindered the integration of digitization content into curricula. 

Although such challenges are identified across the globe, curricula should be designed on a national level to incorporate local health care systems specificities. This echoes Haag et al.’s call for a national initiative “Medical Education in the Digital Age” in Germany [17]. The Medical Faculty Association (MFT) and the Federal Association of Medical Students in Germany (BVMD) have been making efforts to anchor the topic of digitalization in the new medical licensing regulations (ÄAPPO) for some time. Building on such efforts, a unified national initiative would be well-positioned to assist in developing materials and national guidelines and to help faculties better incorporate content on digitization into their curriculum. These efforts may be especially fruitful in fragmented healthcare systems like the one in Germany, where the otherwise large number of interest groups could slow down the digitization process. Additionally, it can be speculated that healthcare systems with many interfaces based on a high level of fragmentation will particularly benefit from digitalization overall.

### 4.1. Limitations

One reason for the divergence between the general belief in the transformative power of digitization and the mild disbelief towards specific, individual digitization trends can be that the faculty representatives define digitization and the associated changes differently and that there is more room for interpretation when it comes to general, unspecific questions. Another limitation of our data is that data collection took place over period of one year due to the methodology, which required appointments with the faculty representatives. Therefore, faculty representatives from the early phase of our data collection may have already undergone curricular changes that we cannot take into account in our study. 

## 5. Conclusion

Our results show that while medical faculties belief in the transformative effect of digitization in health care, there is some disbelief when asked about more specific changes. Our findings also show that the extent to which German universities to prepare students for digitization in health care varies. A majority of faculties miss basic conditions for preparing students for the digital change. 

Due to the COVID-19 pandemic, with its considerable impact on our understanding of location-based work, and also on doctor-patient and doctor-relatives contact, the importance of digitization for medical education will likely increase. For this reason, political organizers and medical faculties need to better prepare students for the upcoming challenges of digital change, for instance by developing uniform guidelines and scenarios that the faculties can use as a basis for their curricular planning. Follow-up studies with health care providers and medical faculties after the COVID-19 pandemic could further investigate such developments.

## Competing interests

The authors declare that they have no competing interests.
